# Fungal Growth in Batch Culture – What We Could Benefit If We Start Looking Closer

**DOI:** 10.3389/fmicb.2019.02391

**Published:** 2019-10-16

**Authors:** Pamela Vrabl, Christoph W. Schinagl, Desirée J. Artmann, Benedikt Heiss, Wolfgang Burgstaller

**Affiliations:** Institute of Microbiology, University of Innsbruck, Innsbruck, Austria

**Keywords:** growth phases, bioreactor batch culture, *Penicillium ochrochloron*, *Aspergillus nidulans*, *Trichoderma harzianum*, respirometry, experimental standardization, alternative growth curve scheme

## Abstract

Since filamentous fungi rapidly adjust their metabolic properties to environmental changes, a rigorous standardization and characterization of cultivation conditions is necessary to obtain meaningful and reproducible results. In batch cultures, which are commonly characterized according to the classical growth curve in textbooks (i.e., lag, exponential, stationary, and declining phase), this is of special difficulty. Although various studies in literature report atypically shaped growth curves of filamentous fungi in batch culture, systematic investigations on this topic are scarce and deviations are barely mentioned in textbooks. Summarizing approximately a decade of observations of growth characteristics from bioreactor batch grown filamentous fungi – in particular two strains (CBS123.823 and CBS123.824) of *Penicillium ochrochloron* – we demonstrate with a series of highly standardized bioreactor batch culture experiments that the classical growth curve failed to describe growth dynamics of the studied fungi in this work. The nature of the first exhausted nutrient was of remarkable importance for the resulting shape of the growth curve. In all experiments, online respirometry proved to be a powerful tool to distinguish growth phases and revealed more physiological states than expected from the mere biomass curve. In this respect we discuss why “atypical” shaped growth curves often remain unrecognized and that they might be the rule rather than the exception. Acknowledging the importance of the correct presentation of this complex topic in textbooks, we also propose a modified growth curve scheme to sensitize students for potential alternative shaped growth curves.

## Introduction

Books must follow sciences, and not sciences books

(Francis Bacon)

This article is a plea for paying more attention to growth conditions and growth curves when growing filamentous fungi in submerged culture. The importance of stating in detail the growth conditions and studying in detail the growth curves of microorganisms grown in batch culture, was clearly stated by Schaechter (2006) and Egli (2015), and is summarized in the statement of Neidhardt et al. (1990) that data derived from “*a poorly characterized culture is all but useless.*” That the transition from one growth phase to another has severe consequences on the macromolecular cell composition, the fluxes through metabolic pathways as well as activity of membrane transport enzymes was documented for bacteria (e.g., Wanner and Egli, 1990; Ferenci, 1999a, b, 2001) and for filamentous fungi (Borrow et al., 1961, 1964). There is, however, much less work for filamentous fungi and the frequency of growth curves deviating from textbook model curves is mostly neglected. Thus the focus of this article is the delimitation and the characterization of the growth phases – and if applicable the physiological states within a growth phase – of our model filamentous fungus *Penicillium ochrochloron* in batch cultures with different nutrient limitations.

**FIGURE 1:**
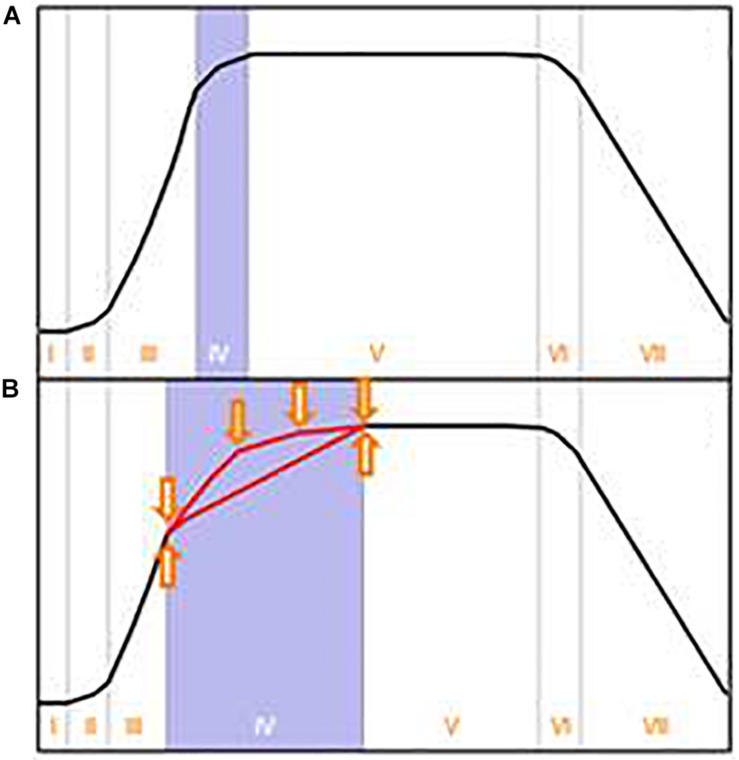
**(A)** Typical growth curve in batch culture as depicted in textbooks. This growth curve, proposed by Buchanan (1918) for bacterial growth in carbon and energy limited cultures, comprises the following growth phases: I- lag, II- transition, III- exponential, IV- transition (also termed deceleration), V- stationary, VI- transition, VII- declining phase. These growth phases are often even more simplified. **(B)** Suggestion for an alternative depiction in textbooks to account for possible variations of the growth curve scheme. Arrows indicate the influence of the first depleted nutrient as well as the importance of the depletion of further nutrients. Adapted and expanded from Buchanan (1918).

It is worth considering the origin of the classical growth curve for batch cultures. As stated in the outstanding review of Wanner and Egli (1990), Buchanan (1918) summarized the knowledge of his time about the dynamics of bacterial growth in batch culture. He proposed a growth curve consisting of seven growth phases, which nowadays is often simplified to the well-known four growth phases: lag, exponential, stationary and declining phase (e.g., Figure 1A). The data on which he based his scheme were derived from heterotrophic bacteria under carbon and energy restricted growth conditions. Although Buchanan’s growth curve was deduced from a very specific experiment, it appears that it was subsequently generalized in textbooks in two respects: First, it was assumed that Buchanan’s growth curve concept holds true for most microorganisms, including organisms with complex intracellular organization and enormous phenotypic plasticity like filamentous fungi. Secondly, it was assumed that growth in batch culture stops immediately after depletion of the first essential nutrient – irrespective of the nature of this essential nutrient.

This inadequate simplification in textbooks and thus often also in teaching has consequences for research. “*What is clear from many of the growth curves published in the literature is that despite the simplicity of appearance and familiarity of the [*…*] batch growth curve, analysis of them is often superficial and the complexity of the processes occurring is then overlooked*” Mason and Egli (1993). Or in other words, up to date dynamics in batch cultures are alarmingly poorly understood (Mason and Egli, 1993).

Deviations from the general growth curves given in textbooks are frequent, although most textbooks seem to consider such deviations – if mentioned at all – as an exception (Supplementary Table S1). Examples for bacteria can be found in Wanner and Egli (1990), and for filamentous fungi in Borrow et al. (1961, 1964) as well as in the literature on organic acid production by *Aspergillus niger* (Chmiel, 1975; Kubicek et al., 1980).

The shape of a growth curve is affected – if not determined – by the nature of the first essential nutrient depleted and by the capability of the organism to cope with its restriction. This has been demonstrated by systematic studies on this subject involving the bacterium *Klebsiella pneumoniae* (Wanner and Egli, 1990) and the fungus *Gibberella fujikuroi* (Borrow et al., 1961, 1964). In both organisms, the first depleted nutrient was decisive of the growth curve’s further progress, and in many cases the growth curves contained a substantially prolonged deceleration phase before entering the stationary phase.

Assuming a high variability of growth curves to be common, why are there not more reports in the literature? In many studies the experimental setup is inadequate to detect complex growth dynamics (Wanner and Egli, 1990). A few examples: (i) if biomass is the only parameter and sampling frequency is low, it is unlikely to detect any deviation from the classical growth curve; (ii) the course of nutrient depletions often remains unknown (Neidhardt et al., 1990) or is limited to the carbon source. The latter is particularly problematic in carbon-excess cultures, where other essential nutrients may exhaust unnoticed and lead to an unrecognized change in growth dynamics as well as physiology; (iii) changes in cultivation conditions (e.g., agitation speed) can conceal a potential deviation; (iv) online measurement of cultivation parameters (e.g., dissolved oxygen tension (DOT), oxygen consumption, proton excretion, or formation of biomass) is often absent, although this would facilitate the identification of nutritional and physiological changes in the culture, as demonstrated later in this study.

A detailed study of growth curves is of special importance for filamentous fungi because of their intrinsic morphological and physiological heterogeneity within hyphae and mycelia (Bleichrodt et al., 2012; Wösten et al., 2013) and their extraordinary phenotypic plasticity/variability elicited by minor changes in their environment (Foster, 1949; Park et al., 2010; Read, 2006).

In this study we summarized approximately a decade of observations of growth characteristics from bioreactor batch grown filamentous fungi – in particular the two *P. ochrochloron* strains CBS123.823 and CBS123.824. These two strains were initially used for metal leaching from industrial wastes (Schinner and Burgstaller, 1989) and developed in the meantime to the second best – besides *A. niger* – investigated species concerning organic acid excretion by filamentous fungi (Vrabl et al., 2017). Experiments were performed in highly standardized bioreactor batch cultures with defined minimal media and with constant temperature, pH and aeration. Besides the nutritional status, also the excretion of organic acids and concentration of biomass in terms of dry weight were estimated with a high sampling frequency. The data were gained during different experimental contexts, by different experimenters and using a variety of bioreactor types (ranging from approximately 2–10 L working volume), all coupled to online respirometry. Thus, the results obtained also highlight the robustness of the observed phenomena.

We noticed (i) that growth of *P. ochrochloron* under conditions leading to organic acid excretion (i.e., glucose excess and ammonium as first nutrient to be depleted) strongly deviated from the textbook growth curve, and (ii) that the exhaustion of ammonium (i.e., end of exponential growth) was associated with a strong decrease in oxygen consumption and carbon dioxide evolution (Vrabl et al., 2008, 2009). Consequently, we payed special attention to this phenomenon while exploring the excretion of organic acids in batch cultures under various nutrient limitations. We focused on the succession of the exponential growth phase to the deceleration phase before the culture enters the stationary phase, as this phase is of utmost importance for many biotechnological applications. Of further interest was the suitability of online respirometry to delimit growth phases quasi-online (especially the exponential growth phase), which would allow a more precise timing of sampling in a defined growth phase.

For *Escherichia coli* studies concerning changes on transcriptomic, proteomic and metabolomic levels related to nutrient exhaustion in batch cultures are available. The situation with filamentous fungi is, however, completely different. Not only are there no such studies for filamentous fungi, but even worse detailed studies of growth curves related to the exhaustion of different nutrient are missing (Meyer et al., 2016). Before “…omics” studies of the effect of nutrient exhaustion in batch culture of filamentous fungi can be carried out meaningfully, detailed knowledge about the growth curves under different nutrient limitations must be available. Therefore the aim of this work is to provide characteristic growth curves of the filamentous fungus *P. ochrochloron* with different nutrient limitations and to characterize the physiological states after nutrient exhaustion in batch cultures using quasi online respirometry.

We consider this work as a contribution to demonstrate the widespread presence of “atypically” shaped growth curves – especially with filamentous fungi. Being additionally involved in teaching and in higher education research and thus acknowledging the importance of the correct presentation of this topic in textbooks, we also propose a modified growth curve scheme to sensitize students for potential alternatively shaped growth curves.

## Materials and Methods

### Fungal Strains

Most experiments were performed with the *Penicillium ochrochloron* strains CBS123.823 and CBS123.824. In addition *Trichoderma harzianum* (kindly donated from Hermann Strasser, University of Innsbruck) and *Aspergillus nidulans* FGSC A4 (kindly donated from Hubertus Haas, Medical University of Innsbruck) were used to study their growth in ammonium limited batch cultivations.

### Precultures

#### Preculture Media

The preculture medium varied depending on the media composition of the main culture. For most bioreactor batch cultivations, where ammonium was used as nitrogen source and glucose as carbon-source, a filamentous growing preculture was produced using a HEPES-glucose medium (Gallmetzer et al., 1998) with the following composition (mM): glucose × 1 H_2_O (400), (NH_4_)_2_SO_4_ (6.25), NH_4_Cl (12.5), KH_2_PO_4_ (5.8), MgSO_4_ × 7 H_2_O (1.6), HEPES (1000), 10 mL L^–1^ trace element solution (see below); pH 7.3 adjusted with 10 M NaOH. For bioreactor batch experiments with nitrate as nitrogen source the preculture medium was as follows (mM): glucose × 1 H_2_O (400), NaNO_3_ (25), NaCl (12.5), KH_2_PO_4_ (5.8), MgSO_4_ × 7 H_2_O (1.6), NaSO_4_ (6.25), HEPES (1000), 10 mL L^–1^ of trace element solution; pH 7.0 adjusted with 10 M NaOH. In case of citrate as carbon source a modified medium after Simkovic et al. (2002) was used which consisted of (mM): citrate × 1 H_2_O (250), (NH_4_)_2_SO_4_ (6.25), NH_4_Cl (12.5), KH_2_PO_4_ (5.8), MgSO_4_ × 7 H_2_O (1.6), 10 mL L^–1^ trace element solution (see below); pH 5.0 adjusted with 10 M NaOH. The trace element solution consisted of (mM): Fe(II)SO_4_ × 7 H_2_O (3.6), Mn(II)SO_4_ × 1 H_2_O (2.8), ZnCl_2_ (2.94), Cu(II)SO_4_ × 5 H_2_O (0.4) and CaCl_2_ × 2 H_2_O (4.0). To avoid precipitation, the pH was adjusted to 3.0 with 5 M HCl. Glucose, salts and – if present – HEPES were sterilized separately and combined aseptically after reaching room temperature. The trace element solution was sterile filtered (0.22 μm, cellulose acetate) and also added aseptically.

#### Incubation

Precultures were grown in 500 mL Erlenmeyer flasks, each containing 100 mL preculture medium, which was inoculated with an appropriate amount of spores to reach a spore density of approximately 10^6^–10^7^ spores per mL. The cultures were incubated at 30°C and 350 rpm with a diameter of rotary motion of 25 mm on an orbitary shaker (Certomat, Braun) for 60–72 h, with the exception of cultures with nitrate as the sole nitrogen source, which grew at a slower rate and were therefore incubated for 75–80 h. Nitrogen was depleted in all precultures after approximately 48 h. At the end of incubation, the precultures were still in the deceleration phase and had not yet reached the stationary phase. The bioreactors were inoculated with 50 mL preculture per L working volume.

### Conditions for Bioreactor Batch Cultivations

#### Bioreactor Batch Media

The medium composition for bioreactor batch cultivations varied depending on the nutrient to be depleted first:

*Glucose-limitation.* The medium consisted of (mM) glucose × 1 H_2_O (20), (NH_4_)_2_SO_4_ (6.25), NH_4_Cl (12.5), KH_2_PO_4_ (5.8), MgSO_4_ × 7 H_2_O (1.6). Cultivations were performed at pH 7.0, which was kept constant with 1 M NaOH.

*Ammonium-limitation (with glucose as carbon source).* The medium (after Vrabl et al., 2012) consisted of (mM) glucose × 1 H_2_O (400), (NH_4_)_2_SO_4_ (5), NH_4_Cl (10), KH_2_PO_4_ (5.8), MgSO_4_ × 7 H_2_O (1.6). Cultivations were performed at pH 5.0 or pH 7.0, respectively, which was kept constant with 1 M NaOH.

*Ammonium-limitation (with citrate as carbon source).* The medium consisted of (mM) citrate × 1 H_2_O (250), (NH_4_)_2_SO_4_ (6.25), NH_4_Cl (12.5), KH_2_PO_4_ (5.8), MgSO_4_ × 7 H_2_O (1.6). Cultivations were performed at pH 5.0, which was kept constant with 1 M HCl and 1 M NaOH (the latter was needed at the end of the cultivation).

*Nitrate-limitation.* The medium consisted of (mM) glucose × 1 H_2_O (400), NaNO_3_ (12.5), NaCl (12.5), KH_2_PO_4_ (5.8), MgSO_4_ × 7 H_2_O (1.6), NaSO_4_ (6.25). Cultivations were performed at pH 7.0, which was kept constant with 1 M NaOH.

*Phosphate-limitation.* The medium (after Vrabl et al., 2012) consisted of (mM) glucose × 1 H_2_O (400), (NH_4_)_2_SO_4_ (6.25), NH_4_Cl (12.5), KH_2_PO_4_ (0.5), KCl (3.8), MgSO_4_ × 7 H_2_O (5.3). Cultivations were performed at pH 5.0 or pH 7.0, respectively, which was kept constant with 1 M NaOH.

In all media 1 g L^–1^ antifoam agent (Clerol FBA 5075, Cognis, Germany) and 10 mL L^–1^ trace element solution were added. Sugar and salts were autoclaved separately and combined aseptically after reaching room temperature. The trace element solution was sterilized by filtration (cellulose acetate filter, pore size 0.22 μm) and also added aseptically.

#### Bioreactor Batch Cultivations

Bioreactor batch cultivations were performed either in bioreactors with 1.8 L working volume (Biostat M or Biostat A bioreactor; Sartorius/Braun, Melsungen, Germany), KLF 2000 bioreactor (Bioengineering; Wald, Switzerland), with 4.5 L working volume (Biostat B; Sartorius/Braun, Melsungen, Germany) or 10 L working volume (NLF 200; Bioengineering, Wald, Switzerland). All bioreactors were operated with identical stirrer types with identical stirrer circumferential speed and therefore equal shearing forces. The experiments were carried out at 30°C, 700–1300 rpm and 0.56 vvm (volume of air per volume of medium and minute; 1 l^–1^ min^–1^). The further details of sampling and sample treatment followed the procedure as previously described (Vrabl et al., 2012). For the bioreactors with small working volumes, only one sample was taken per sampling time in order to disturb the cultivation and experimental conditions as little as possible. At least three independent bioreactor batch cultivations were performed for each nutrient condition investigated. Additionally, at least one undisturbed control cultivation (i.e., without sampling) was carried out per condition. Although the growth curves in the parallel experiments were similar, we observed slight temporal shifts caused, for example, by a slightly different inoculum and/or different bioreactors. To take into account the inter-bioreactor variability, the data from different experiments of more than 20–40 replicate batch cultivations were not averaged, but one representative experiment is shown for each nutrient limitation.

#### Delimitation of Growth Phases

The end of the lag phase (i.e., the beginning of exponential growth) was approximated by determining the intercept of the logarithmic plot of the oxygen uptake curve with the x-axis of the initial oxygen uptake level. The end of exponential growth was indicated by the depletion of the first limiting nutrient, which was also reflected in the respiration curves (see section Looking Closer With Online Respirometry – A Powerful Tool to Improve Experimental Standardization of Batch Cultures). A clear delimitation between the deceleration phase and the stationary phase was difficult and was a trade-off between biomass and respiratory markers (see section Looking Closer With Online Respirometry – A Powerful Tool to Improve Experimental Standardization of Batch Cultures).

### Meta-Analysis of the Culture Media Composition

Egli (2015) emphasized that quantitative aspects of the media composition should be given greater attention in order to avoid the use of inappropriate media. One of these aspects is that it should be proved which nutrient limits growth stoichiometrically and whether the other nutrients are in sufficient excess (recommended excess factors in a carbon limited medium are 3–5 for N, 5–10 for P, S, K. Mg and 10–20 for Fe; Egli, 2015). Following this suggestion, we analyzed the media used for *P. ochrochloron* in this work according to the procedure exemplified in Tables 1, 2 of Egli (2015). In addition, we have partially experimentally verified the calculated results.

Central for the calculation of excess factors are the values for the biomass yield (Y_X/E_; g dry weight/g element). The Y_X/E_ values strongly depend on the composition of the medium, other cultivation conditions and the specific growth rate. Therefore, we did not use the YX/E values given by Egli (2015), derived from gram negative bacterial cells growing at μmax in batch culture, but Y_X/E_ values calculated from a series of batch cultivations with *P. ochrochloron*. In general, our own Y_X/E_ values for C, N, and P are higher than those of Egli (2015). Reasons for this could be for instance the heterotrophic carbon dioxide fixation (in the case of carbon limited growth; the respiratory quotient in these cultivations was between 0.6 and 0.9 and thus distinctly below 1) or the accumulation of reserve carbohydrates (in the case of nitrogen limited growth). Values of Y_X/E_ higher than 1 were also found for other filamentous fungi cultivated in bioreactor batch culture at near neutral pH (Supplementary Table S2).

**FIGURE 2:**
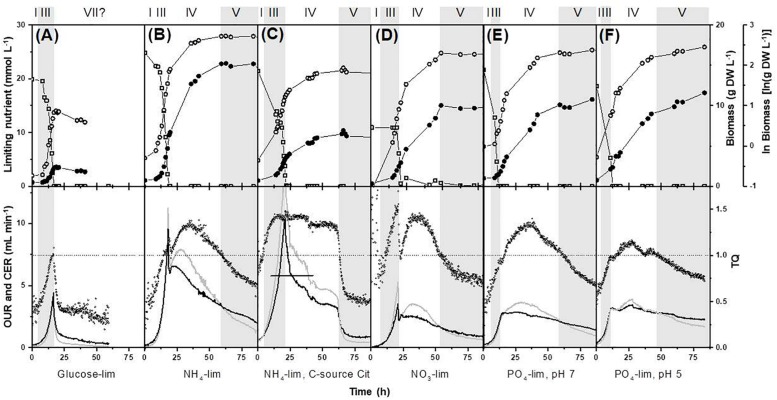
Effect of different nutrient limitation in a defined minimal medium on biomass evolution, nutrient uptake, oxygen consumption and carbon dioxide production on bioreactor batch cultivations of *Pencillium ochrochloron* CBS123.823 carried out in bioreactors with 1.8 L working volume. The limiting nutrient was **(A)** glucose, **(B,C)** ammonium, **(D)** nitrate, **(E)** phosphate at pH 7, and **(F)** phosphate at pH 5. All cultures except for **(C)** NH_4_-Cit were grown with glucose as carbon-source. The original carbon dioxide data in **(C)** above the vertical line were extrapolated with other parallel experiments as the carbon dioxide sensor in this experiment was saturated. Later experiments with this condition used a less sensitive sensor. Delimitation of growth phases were done as described in section “Materials and Methods.” Numbering of growth phases are according to Figure 1: I, lag phase; III, exponential phase; IV, deceleration phase; V, stationary phase; VII, declining phase. Upper panel, limiting nutrient (open squares), biomass (closed circles), ln(biomass) (open circles); lower panel: OUR, oxygen uptake rate (gray line); CER, carbon dioxide evolution rate (black line); TQ, transfer quotient calculated as CER/OUR (small open circles) (Royce, 1992). Note that some datasets depicted here or in Supplementary Files, namely in **(B,E,F)** have been expanded from previous works (Vrabl et al., 2009, 2012). Representative experiments are shown in this figure and further examples in Supplementary Figure S1.

The calculated excess factors for the media used are given in Supplementary Table S3. These excess factors indicate that the growth of *P. ochrochloron* was indeed limited by a single nutrient when grown in carbon, nitrogen or phosphorus limited batch culture. This was confirmed experimentally by measuring glucose, ammonium and phosphate in the culture filtrates (Figure 2).

However, the results given in Supplementary Table S3 suggest a growth limitation by calcium and iron. We have tested this prediction in shake flask cultures (Supplementary Table S4). Neither additional supplementation with iron nor an elevated concentration of trace element solution increased the formation of biomass. This contradiction between calculated and experimental results may perhaps be due to inappropriate yield factors (assuming that oxygen was not limiting growth in shake flask cultures) or due to the insolubility of the additionally added trace elements at a pH near 7.

### Analytical Methods

Glucose, ammonium, phosphate and extracellular organic acids in culture filtrates, dry weight, oxygen consumption and carbon dioxide production were determined as previously described (Vrabl et al., 2012). Nitrate was measured by HPLC using a Hypersil ODS 5 μ precolumn (40 × 4.6 mm, ARC Seibersdorf, Austria) using the method of Doblander and Lackner (1996).

## Results and Discussion

### Growth Curves of *Penicillium ochrochloron* and Other Filamentous Fungi Depending on the Nature of the First Exhausted Nutrient

**FIGURE 3:**
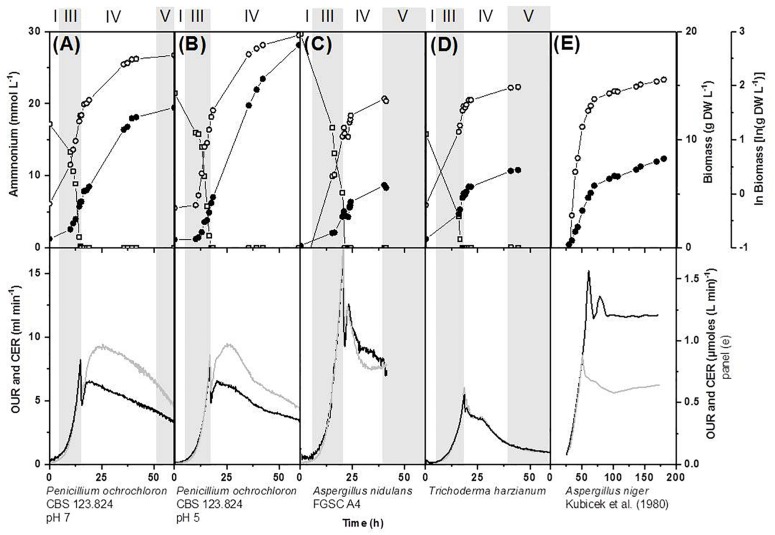
Effect of ammonium limitation in a defined medium on biomass evolution, nutrient uptake, oxygen consumption and carbon dioxide production on bioreactor batch cultivations of various filamentous fungi carried out in bioreactors with 1.7 L working volume **(A,B,D)** and 4.5 L working volume **(C)**. **(A,B)** *Pencillium ochrochloron* CBS123.824 cultivated at pH 7 and pH 5, **(C)** *Aspergillus nidulans* FGSC A4 and **(D)** *Trichoderma harzianium*. Data for **(E)** *Aspergillus niger* were derived from Kubicek et al. (1980) by extracting the data from the respective figures with the software “Engauge Digitizer Version 4.1” and replotting them. Delimitation of growth phases were done as described in section “Materials and Methods.” Numbering of growth phases are according to Figure 1: I, lag phase; III, exponential phase; IV, deceleration phase; V, stationary phase; VII, declining phase; OUR, oxygen uptake rate; CER, carbon dioxide evolution rate. Upper panel: limiting nutrient (open squares), biomass (closed circles), ln(biomass) (open circles). Lower panel: OUR, oxygen uptake rate (gray line); CER, carbon dioxide evolution rate (black line). Note that some datasets depicted here or in Supplementary Files, namely in **(A,B)** have partly been rearranged and expanded from a previous work (Vrabl et al., 2012). Representative experiments are shown in this figure and further examples in Supplementary Figure S2.

None of the growth curves observed in our experiments followed the progress of the classical growth curve. Additionally, the nature of the first depleted nutrient in *P. ochrochloron*, grown over a broad range of various nutrient limitations, strongly influenced the further progress of the growth curve (Figures 2, 3).

When glucose was the first exhausted nutrient, the culture passed from the exponential growth phase to an extremely short, almost negligible stationary phase, followed by a decline in dry weight (Figure 2A). Unsurprisingly, the onset of glucose exhaustion caused strong metabolic responses, for instance, an immediate decrease of respiration rates (more details on respirometry to delimit growth phases see section Looking Closer With Online Respirometry – A Powerful Tool to Improve Experimental Standardization of Batch Cultures) and the immediate reuptake of previously excreted organic acids. These findings are not specific to *P. ochrochloron* as studies of other filamentous fungi report comparable observations (e.g., Larsen et al., 2004; Diano et al., 2006; Dowdells et al., 2010; Schmitz et al., 2013). Also morphological alterations under glucose or carbon starvation such as progressive vacuolation are known (e.g., Paul et al., 1994; Harvey et al., 1998; Pollack et al., 2008). This would explain why carbon starved cultures of filamentous fungi frequently decline in dry weight (e.g., Borrow et al., 1961; Carter and Bull, 1969; Trinci and Righelato, 1970; Larmour and Marchant, 1977).

In contrast to glucose limitation, the exponential growth phase was followed by a considerably prolonged deceleration phase in all phosphate and nitrogen limited cultivations of *P. ochrochloron*, *T. harzianum*, and *A. nidulans* (Figures 2, 3). Also in these experiments the end of the exponential growth phase was clearly indicated by the respiration curves (more details see section Looking Closer With Online Respirometry – A Powerful Tool to Improve Experimental Standardization of Batch Cultures) and the biomass continued to increase after depletion of the first essential nutrient. The length of the deceleration phase and the increase in dry weight depended on the nature of the first nutrient depleted as well as the carbon source and was especially pronounced in phosphate limited cultures (up to sixfold increase in dry weight). As we will discuss in more detail later (see section Looking Even More Closer – The Transition Phase Between Exponential Growth Phase and Stationary Growth Phase), the observation of a prolonged deceleration phase was not a unique characteristic of the fungal species examined in this work, but appears to be present in many other microorganisms.

### Looking Closer With Online Respirometry – A Powerful Tool to Improve Experimental Standardization of Batch Cultures

Respirometry is frequently used to determine a broad range of parameters related to growth in batch cultures (Goudar and Strevett, 1998; Garcia-Ochoa et al., 2010). Considering the key function of oxygen in aerobic (micro)organisms, the oxygen uptake rate (OUR) in cultures without oxygen limitation can be used as an indicator for metabolic activity and to optimize growth conditions for improved product yields (Garcia-Ochoa et al., 2010). It is evident that a correct delimitation of the growth phases in batch cultures, especially of the exponential growth phase, is paramount considering the rapidly occurring changes in physiology. Therefore the suitability of respiration curves to do this was of special interest in this work.

In all our experiments, the end of exponential growth was clearly indicated in the respiration curves (Figures 2, 3) or – when available – in the corresponding dissolved oxygen curve. This observation was highly reproducible for all tested filamentous fungi. The respiration curves also indicated drastic shifts in catabolic fluxes in the subsequent deceleration phase (see also section Looking Even More Closer – The Transition Phase Between Exponential Growth Phase and Stationary Growth Phase), though most dynamics within the respiration curves still remained unknown. An exception was when the carbon source in carbon excess cultures was exhausted, which could usually be traced back in the corresponding respiration curve (e.g., strong decrease in respiration in Figure 2C at approximately 60 h is due to citrate reaching low levels and becoming depleted at approximately 64 h of cultivation time).

In contrast to the clear delimitation of the exponential growth phase, there was no comparable strong marker which indicated the start of the stationary phase. We found, however, that the beginning of the stationary phase often, but not in all experiments (e.g., not in Figure 2F), was given when a transfer quotient (TQ; Royce, 1992) of 1 was reached again (Figures 2B–E). However, this could be a coincidence for cultures at pH 7 and might not hold true for cultures that have grown at other pH levels due to the pH-sensitive carbon dioxide equilibrium.

Remarkably, each limiting nutrient seemed to trigger a specific pattern in the respiration curves (e.g., compare respiration of glucose, ammonium, and phosphate limitation, Figure 2). Furthermore, there is evidence that these progresses might be species-independent. For example, the characteristic double peaked respiration curves for *P. ochrochloron, T. harzianum, A. nidulans* (present work Figures 2B,D, 3A–D) and *A. niger* (Kubicek et al., 1980, Figure 3E) were strikingly similar at the onset of a nitrogen limitation when glucose was the carbon source. DOT curves of ammonium-limited grown *E. coli* also showed a double peak (Brethauer et al., 2007), which was even more pronounced than those observed in this study. In contrast to nitrogen limitation, glucose limitation triggered a single-peak response in *P. ochrochloron* (Figure 2A), which was also reported for DOT curves of *P. chrysogenum* (Goudar et al., 1999) or *Aspergillus oryzae* (Larsen et al., 2004). We therefore hypothesize that, similarly to the progress of the growth curves, the nature of the first nutrient depleted is decisive for the shape of the corresponding respiratory curve. These characteristic respiratory patterns might be similar for a broad range of aerobic microorganisms.

Summarizing the above mentioned observations, we think that the potential of respirometry to characterize growth characteristics and physiological in submerged cultures of filamentous fungi states is far from being exhausted and is definitely worth to pay more attention to it.

### Looking Even More Closer – The Transition Phase Between Exponential Growth Phase and Stationary Growth Phase

As already mentioned, in most of our experiments the duration of the deceleration phase was considerably prolonged and exceeded the length of the exponential growth phase (Figures 2, 3). These findings are not restricted to the filamentous fungi examined in this work, but are also in line with a wealth of growth curves of even very well-investigated microorganisms like *Klebsiella pneumoniae* (Wanner and Egli, 1990) or *E. coli* (Baev et al., 2006), *Saccharomycopsis lipolytica* (Briffaud and Engasser, 1979; Klasson et al., 1989; Moresi, 1994), *Aspergillus nidulans* (Dorn and Rivera, 1966; Trinci, 1970), *Aspergillus niger* (Chmiel, 1975; Kubicek and Röhr, 1977; Kubicek et al., 1980; Röhr et al., 1981; Dawson and Maddox, 1988; Mesojednik and Legisa, 2005), *Aspergillus foetidus* (Kristiansen and Sinclair, 1978), *G. fujikuroi* (Borrow et al., 1961, 1964), *Neurospora crassa* (Gillie, 1968), *or Penicillium chrysogenum* (Trinci, 1970) which all show this type of “deviation.” The overwhelming evidence from the research literature severely contrasts the concept of a short, even negligible deceleration phase as often depicted in textbooks or other teaching materials (Mason and Egli, 1993). Thus, we concur with Mason and Egli (1993), who suggested that the nature of the first exhausted nutrient is relevant for the further progress of the deceleration phase, which is also reflected in the characteristic progress of the respirometric curves in our own experiments (section Looking Closer With Online Respirometry – A Powerful Tool to Improve Experimental Standardization of Batch Cultures). Moreover, there is evidence from literature that the sequence of further nutrient depletions also strongly determines the shape and length of the deceleration phase (Borrow et al., 1961, 1964).

**FIGURE 4:**
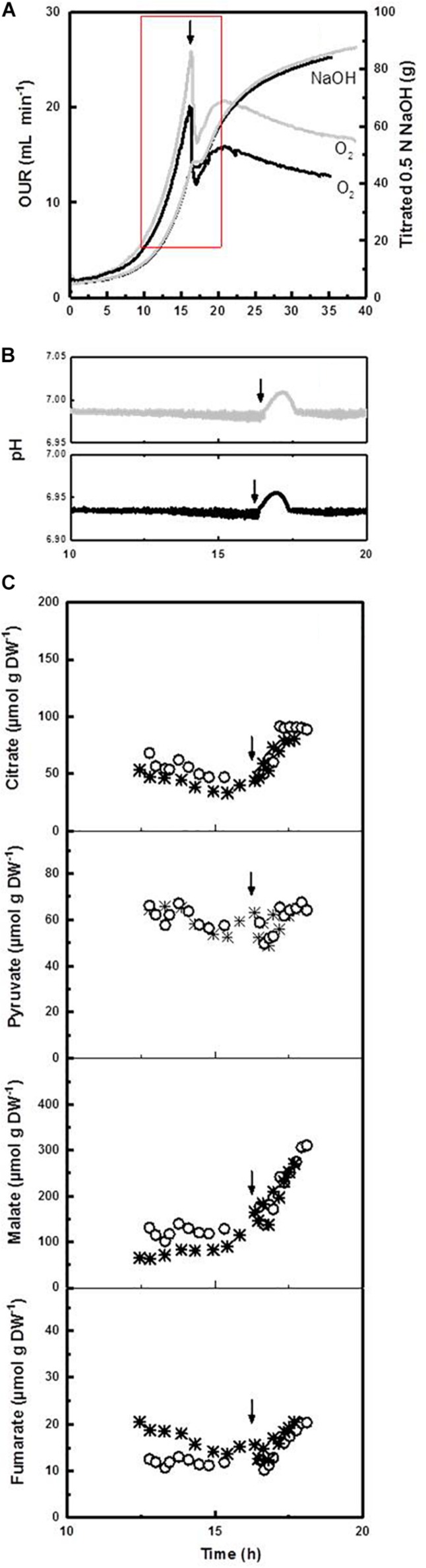
Increased time resolution experiments exploring the transition between exponential growth phase and the deceleration phase of ammonium limited cultures at pH 7 of *Penicillium ochrochloron* CBS123.824 illustrated with two independent cultivations. Experiments were carried out in a Biostat B bioreactor with 5 L working volume. The arrow indicates the time point of ammonium exhaustion in the culture. Batch #1 (**○**), batch #2 (*). **(A)** Oxygen uptake rate (OUR) and amount of titrated NaOH (0.5 N) over the whole cultivation. Batch #1 (gray line), batch #2 (black line). The red square indicates the time frame depicted in **(B,C)**. **(B)** Progress of extracellular pH during the transition from the exponential growth phase and the deceleration phase. Note that cultures at pH 5 with the same strain (depicted in Vrabl et al., 2012) showed an even more distinct rise in extracellular pH over a prolonged time. **(C)** Progress of excreted organic acids and their partial reuptake. Note that the reuptake in cultures at pH 5 with the same strain (depicted in Vrabl et al., 2012) was even more pronounced.

Beside a considerable prolongation, we observed that the deceleration phase is physiologically (and morphologically) a remarkably dynamic growth phase. For instance, in experiments with a high sampling frequency, we found that – apart from the strong response in respirometry – the onset of an ammonium limitation apparently stopped for a short period of time the proton pumping activity of the plasma membrane H^+^-ATPase, which is the main source of ATP driven proton efflux in yeasts and filamentous fungi including *P. ochrochloron* (Vrabl et al., 2012; Figure 4). This pause was accompanied by a quick rise in external pH (Figure 4) and probably also a short stop in biomass production. In a previous work (Vrabl et al., 2008) we furthermore noticed that in this phase also a drastic change in glucose uptake rate occurs, which we also found in other fungi after analyzing published data of *A. niger*, *A. awamori*, *G. fujikuroi*, and *S. lipolytica*.

Interestingly, the length and progress of the observed rise in pH was strain and pH dependent and ranged from a few hours at pH 7 (Figure 4) to a period of at least 40–60 h at pH 5 (Vrabl et al., 2012). In a previous work we hypothesized that this rise was due to an observed reuptake of organic acids despite glucose excess conditions, indicating a probably still unknown function of organic acid excretion (Vrabl et al., 2012). These results unequivocally document that the deceleration phase is a period of major metabolic rearrangements which regard several different levels of metabolism.

Considering the severe physiological alterations caused by nutrient exhaustion (Mason and Egli, 1993), these findings of a dynamic deceleration phase are not very surprising. Nevertheless “*the many and varied factors governing entry into, and growth during the deceleration phase have been given little attention. Growth kinetics during this period are therefore largely uncharacterized, despite the importance of the deceleration phase for biotechnological processes, as the period when secondary metabolite production begins*” (Prosser, 1994). It appears that more than 20 years later this statement is still true and many important physiological processes occurring in this growth phase have yet to be explored.

### Factors Influencing the Shape of Growth Curves – A Short Summary

**FIGURE 5:**
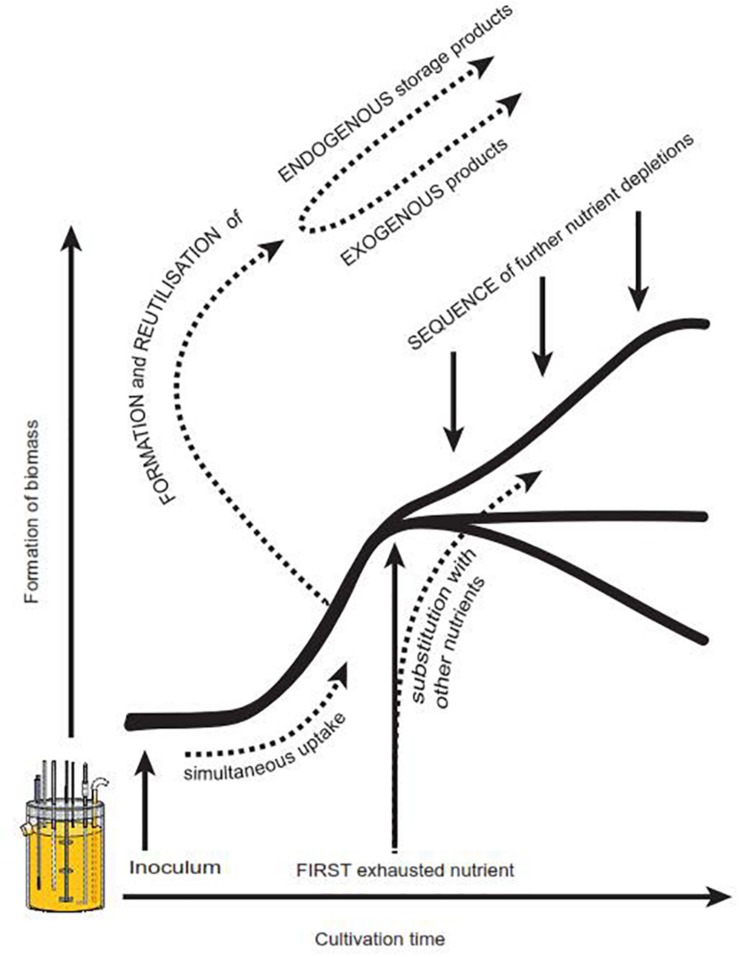
Factors known to influence the progress of a growth curve. Further details see also section Factors Influencing the Shape of Growth Curves – A Short Summary.

So far, a number of factors have been identified in literature which evidently influence the progress of a microbial growth curve, but have not yet been recognized for their importance in textbooks and teaching material. We thus strongly suggest a modified growth curve scheme (Figure 1B) to increase students’ awareness of potential alternatively shaped growth curves. In addition, we propose Figure 5, which offers a brief summary of what we consider to be the most relevant influencing parameters: There is strong evidence that the nature of the first exhausted nutrient is of remarkable importance for the resulting shape of a growth curve (Borrow et al., 1961, 1964; Wanner and Egli, 1990; Mason and Egli, 1993). Also the sequence of all subsequent nutrient depletions, especially the time of glucose exhaustion, influences the further progress of a growth curve (Borrow et al., 1961, 1964). Another source for deviations from the classical standard growth curve is hidden diauxic growth, which can occur via reutilization of previously excreted metabolites such as polyols (Röhr et al., 1987; Diano et al., 2006), gluconate (Schmitz et al., 2013) or organic acids (Larsen et al., 2004; Andersen et al., 2009; Dowdells et al., 2010; present work). In case nutrients serve similar metabolic functions (e.g., potassium and sodium, or, different carbon sources), they may replace or complement each other (Jones and Gadd, 1990; Weusthuis et al., 1994; Zinn et al., 2004). Depending on the nature of these nutrients and their relative concentration within the medium, typical diauxic growth or simultaneous nutrient uptake can occur (Law and Button, 1977; Wanner and Egli, 1990; Kovarova-Kovar and Egli, 1998; Rodriguez-Navarro, 2000; Zinn et al., 2004; Schmitz et al., 2013). Finally, the inoculum with its cultural history and thus its physiological state, morphology, cell density or volumetric size has a considerable influence on the further progress of a batch culture, including its productivity (Meyrath, 1962; Meyrath and Mcintosh, 1963; Mcintosh and Meyrath, 1963; Law and Button, 1977; Mason and Egli, 1993; Paul et al., 1999; Schaechter, 2006; Ryall et al., 2012) In addition, in bacteria the properties of the inoculum appear to have direct consequences on the progress of the declining phase (Phaiboun et al., 2015).

## Conclusion

Based on literature data and summarizing over a decade of our own work, we are in line with others and hypothesize that growth curves of a non-textbook type are the rule rather than the exception for filamentous fungi, as seems to be the case for many other microorganisms. Despite being a fundamental method in microbiology, growth and growth curves are still vastly underrepresented in textbooks. Moreover, the frequent oversimplification of the growth curve itself leads to a general misconception of its complexity and to an undervaluation of the often extreme, largely unexplored physiological dynamics the cultures undergo. This in turn has a direct and strong consequence on all experimental aspects. As long as “*microbial growth seems to be considered as a ‘specialised subject’, a point that has little relevance to the question that one wants to investigate (or, alternatively, one assumes that it is safe to follow ‘standard’ or often used procedures)*” (Egli, 2015), scientific progress is hampered to a certain extent.

This issue is clearly not a simple academic problem. Considering some of the future challenges for humanity involving filamentous fungi (or microorganisms in general) such as the production of chemical building blocks out of renewable resources (Sauer et al., 2008) or the search for novel antibiotics to combat (multiple) antibiotic resistance (Brakhage and Schroeckh, 2011), the study and understanding of growth and its underlying physiology might be essential to understand these challenges and problems and to find solutions.

## Data Availability Statement

All datasets generated for this study are included in the manuscript/Supplementary Files.

## Author Contributions

PV conceived the overall study and designed it together with BH, CS, and WB. BH, CS, DA, PV, and WB performed the experiments. PV wrote the first draft of the manuscript, which was then critically revised and rewritten by CS, DA, PV, and WB. PV and CS performed the literature research for the text books. DA and WB analyzed the media on the meta-level. All authors contributed to the drafting of the figures, data analysis and interpretation, and read and approved the final manuscript version.

## Conflict of Interest

The authors declare that the research was conducted in the absence of any commercial or financial relationships that could be construed as a potential conflict of interest.
