# Supplementary material for: Fungal Growth in Batch Culture – What We Could Benefit If We Start Looking Closer
Source: Front Microbiol. 2019 Oct 16;10:2391. doi: 10.3389/fmicb.2019.02391 (PMC6805767; doi:10.3389/fmicb.2019.02391)
Supplement: Supplementary file 1 [file Data_Sheet_1.docx]

Supplementary Material

Fungal growth in batch culture - what we could benefit if we start looking closer

Pamela Vrabl*, Christoph W. Schinagl, Desirée J. Artmann, Benedikt Heiss, Wolfgang Burgstaller

*** Correspondence:** Pamela Vrabl: Pamela.Vrabl@uibk.ac.at

**Supplementary Table 1.** Overview of mentioned growth parameters in textbooks.

Abbreviations: tu, topic unmentioned;

* Righelato R.C. (1975). Growth kinetics of mycelial fungi. In: Smith, J.E., Berry, D.R. (Eds.), The Filamentous Fungi, Vol. 1, Industrial Mycology, London: Edward Arnold, 79-103.

** Bull, A.T. (1974). Microbial Growth. In: Bull, A.T., Lagnado, J.R., Thomas, J.O., Tipton K.F. (Eds.) Companion to Biochemistry: Selected Topics for Further Study. London: Longman, chapter 13.

**Supplementary Table 2.** Yield factors for biomass (Y_X/E_; g DW/g C) for carbon limited growth of filamentous fungi in bioreactor batch culture.

| Species | Y_X/E_ (g DW/g C) | Reference |
| --- | --- | --- |
|  |  |  |
| *Penicillium ochrochloron* | 1.66 | This work |
| *Penicillium chrysogenum* | 1.28 | Nielsen (1995) PhD thesis, p 105 |
| *Aspergillus niger* | 1.40 | Nitsche et al., 2012 |
|  | 1.15 | Pedersen et al., 2000 |
| *Aspergillus nidulans* | 1.72 | Carter and Bull, 1969 |
| *Aspergillus oryzae* | 1.25 | Morkeberg et al., 1995 |

**Supplementary Table 3.** Analysis of the media used for growing *Penicillium ochrochloron* nutrient-terminated (Mason and Egli 1993) for carbon, nitrogen and phosphorus in bioreactor batch culture at pH 7.

| Element | Amount element (g/L) | Yield factor (g DW/g element) | DW predicted (g/L) | Excess factor |
| --- | --- | --- | --- | --- |
| **C limited growth (glucose)** |  |  |  | **over C** |
| C | 1.44 | 1.6 | 2.31 | 1.0 |
| N | 0.35 | 12 | 4.22 | 1.8 |
| P | 0.18 | 114 | 20.50 | 8.9 |
| S | 0.28 | 100 | 28.07 | 12.2 |
| K | 0.17 | 100 | 16.75 | 7.3 |
| Mg | 0.08077 | 200 | 16.15 | 7.0 |
| Ca | 0.00162 | 100 | 0.16 | 0.1 |
| Fe | 0.00199 | 200 | 0.40 | 0.2 |
| Mn | 0.00148 | 10000 | 14.80 | 6.4 |
| Zn | 0.00190 | 10000 | 19.00 | 8.2 |
| Cu | 0.00025 | 10000 | 2.52 | 1.1 |
|  |  |  |  |  |
| **N limited growth (NH_4_)** |  |  |  | **over N** |
| C | 28.83 | 1.6 | 46.12 | 18.2 |
| N | 0.21 | 12 | 2.54 | 1.0 |
| P | 0.18 | 114 | 20.50 | 8.1 |
| S | 0.19 | 100 | 18.66 | 7.4 |
| K | 0.23 | 100 | 22.70 | 8.9 |
| Mg | 0.08000 | 200 | 16.15 | 6.4 |
| Ca | 0.00162 | 100 | 0.16 | 0.1 |
| Fe | 0.00199 | 200 | 0.40 | 0.2 |
| Mn | 0.00148 | 10000 | 14.80 | 5.8 |
| Zn | 0.00180 | 10000 | 19.00 | 7.5 |
| Cu | 0.00025 | 10000 | 2.52 | 1.0 |
|  |  |  |  |  |
| **P limited growth (PO_4_)** |  |  |  | **over P** |
| C | 28.83 | 1.6 | 28.83 | 15.9 |
| N | 0.35 | 12 | 4.22 | 2.3 |
| P | 0.02 | 114 | 1.82 | 1.0 |
| S | 0.28 | 100 | 28.07 | 15.5 |
| K | 0.12 | 100 | 11.96 | 6.6 |
| Mg | 0.08000 | 200 | 16.15 | 8.9 |
| Ca | 0.00162 | 100 | 0.16 | 0.1 |
| Fe | 0.00199 | 200 | 0.40 | 0.2 |
| Mn | 0.00148 | 10000 | 14.80 | 8.2 |

| **Supplementary Table 4.** Influence of **trace element concentration** on biomass formation in shake flask cultures of *Penicillium ochrochloron* | | | | | | | | |
| --- | --- | --- | --- | --- | --- | --- | --- | --- |
|  |  |  |  | |  |  |  |  |
| Cultivation time (h) |  | Biomass (g dry weight per Liter) | | | |  |  |  |
|  |  |  |  |  | |  |  |  |
|  |  | **Without trace elements** | **Standard** | **Double** | | **Fivefold** |  |  |
|  |  |  |  |  | |  |  |  |
| **0** |  | 0.11 | 0.14 | 0.15 | | 0.18 |  |  |
| **24** |  | 0.18 | 0.26 | 0.25 | | 0.38 |  |  |
| **48** |  | 1.63 | 2.42 | 2.00 | | 2.92 |  |  |
| **72** |  | 3.20 | 8.03 | 7.09 | | 9.00 |  |  |
| **96** |  | 3.11 | 12.96 | 11.04 | | 10.94 |  |  |
|  |  |  |  |  | |  |  |  |
|  |  | (values are the mean of three parallels) | | | | |  |  |

**
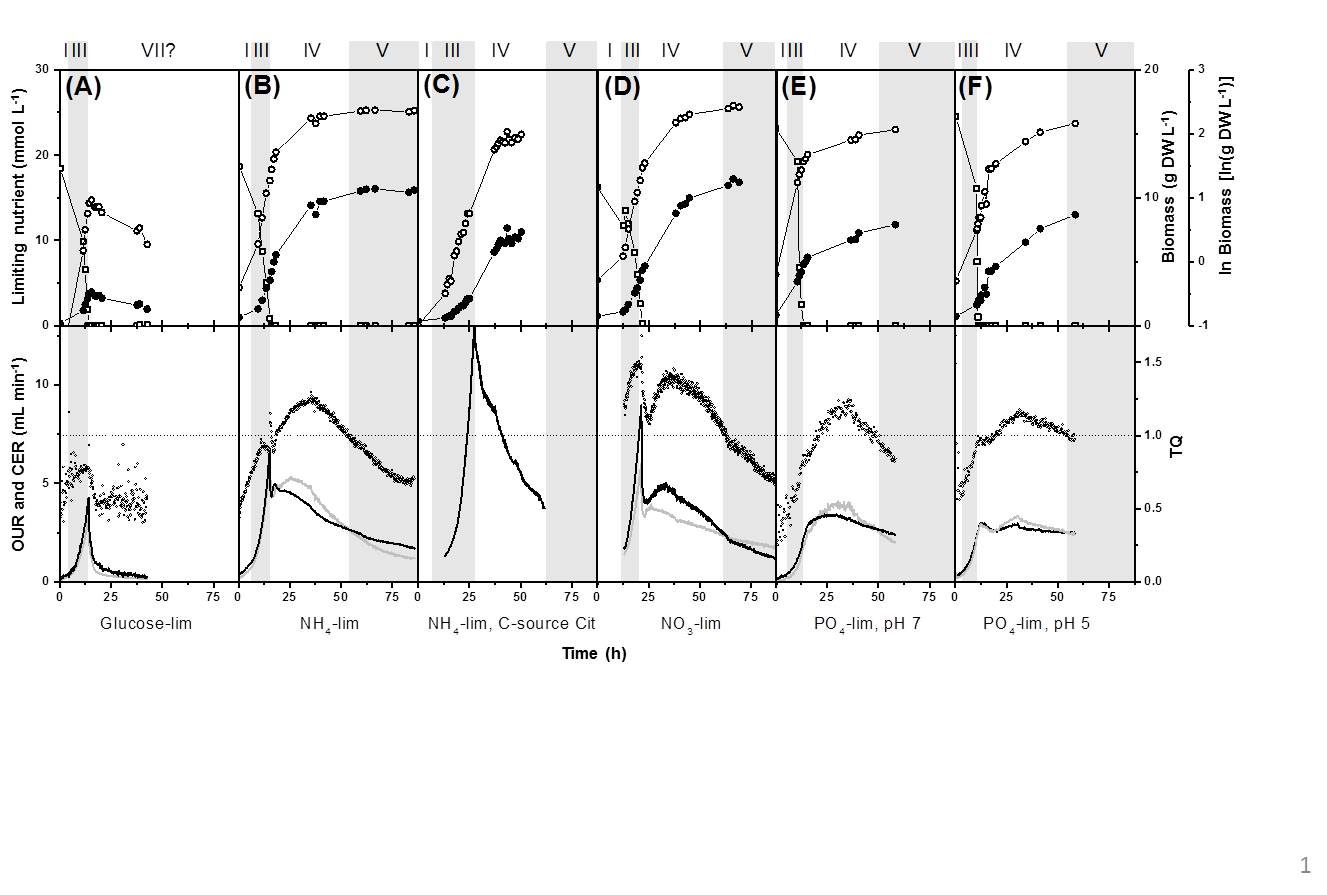
**

**Supplementary Figure 1.** Further examples for the effect of different nutrient limitation in a defined minimal medium on biomass evolution, nutrient uptake, oxygen consumption and carbon dioxide production on bioreactor batch cultivations of Pencillium ochrochloron CBS123.823 carried out in bioreactors with 1.8 L working volume. The limiting nutrient was (A) glucose, (B, C) ammonium, (D) nitrate, (E) phosphate at pH 7 and (F) phosphate at pH 5. All cultures except for (C) NH_4_-Cit were grown with glucose as carbon-source. Delimitation of growth phases were done as described in the section ‘Materials and Methods’. Numbering of growth phases are according to Figure 1: I … lag phase, III … exponential phase, IV … deceleration phase, V … stationary phase, VII … declining phase. Abbrevations and legends: upper panel: limiting nutrient (open squares), biomass (closed circles), ln(biomass) (open circles). lower panel: OUR … oxygen uptake rate (grey line), CER … carbon dioxide evolution rate (black line), TQ … technical quotient calculated as CER/OUR (small open circles).

**
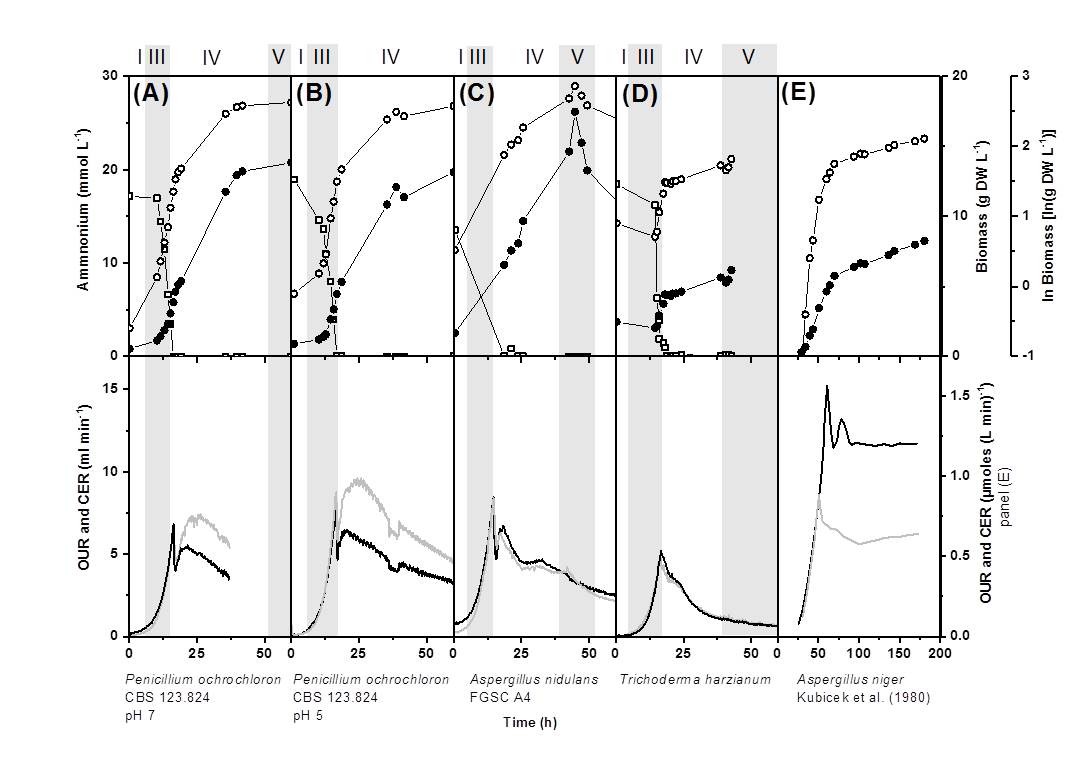
**

**Supplementary Figure 2.**Further examples for the effect of ammonium limitation in a defined medium on biomass evolution, nutrient uptake, oxygen consumption and carbon dioxide production on bioreactor batch cultivations of various filamentous fungi carried out in bioreactors with 1.7 L working volume. **(A, B)** *Pencillium ochrochloron* CBS123.824 cultivated at pH 7 and pH 5, **(C)** *Aspergillus nidulans* FGSC A4 and **(D)** *Trichoderma harzianium*. Data for **(E)** *Aspergillus niger* Figure 3 of the main manuscript in comparison. Delimitation of growth phases were done as described in the section ‘Materials and Methods’. Numbering of growth phases are according to **Figure 1**: I … lag phase, III … exponential phase, IV … deceleration phase, V … stationary phase, VII … declining phase, OUR … oxygen uptake rate, CER … carbon dioxide evolution rate. **Abbrevations and legends**: upper panel: limiting nutrient (open squares), biomass (closed circles), ln(biomass) (open circles). lower panel: OUR … oxygen uptake rate (grey line), CER … carbon dioxide evolution rate (black line).

**
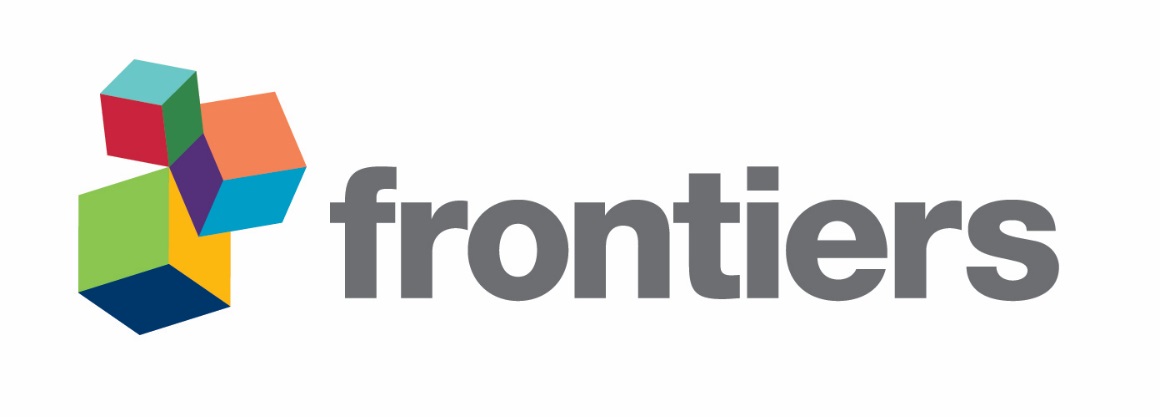
**
